# Organoids of the female reproductive tract

**DOI:** 10.1007/s00109-020-02028-0

**Published:** 2021-02-13

**Authors:** Cindrilla Chumduri, Margherita Y. Turco

**Affiliations:** 1 Department of Microbiology, University of Würzburg, Biocenter, Würzburg, Germany; 2 Max Planck Institute for Infection Biology, Berlin, Germany; 3 Department of Pathology, University of Cambridge, Cambridge, UK; 4 Centre for Trophoblast Research, Cambridge, UK

**Keywords:** Female reproductive tract, Organoids, Reproductive health, Pregnancy, Fertility, Infection, Cancers

## Abstract

Healthy functioning of the female reproductive tract (FRT) depends on balanced and dynamic regulation by hormones during the menstrual cycle, pregnancy and childbirth. The mucosal epithelial lining of different regions of the FRT—ovaries, fallopian tubes, uterus, cervix and vagina—facilitates the selective transport of gametes and successful transfer of the zygote to the uterus where it implants and pregnancy takes place. It also prevents pathogen entry. Recent developments in three-dimensional (3D) organoid systems from the FRT now provide crucial experimental models that recapitulate the cellular heterogeneity and physiological, anatomical and functional properties of the organ in vitro. In this review, we summarise the state of the art on organoids generated from different regions of the FRT. We discuss the potential applications of these powerful in vitro models to study normal physiology, fertility, infections, diseases, drug discovery and personalised medicine.

## Introduction

**Fig. 1:**
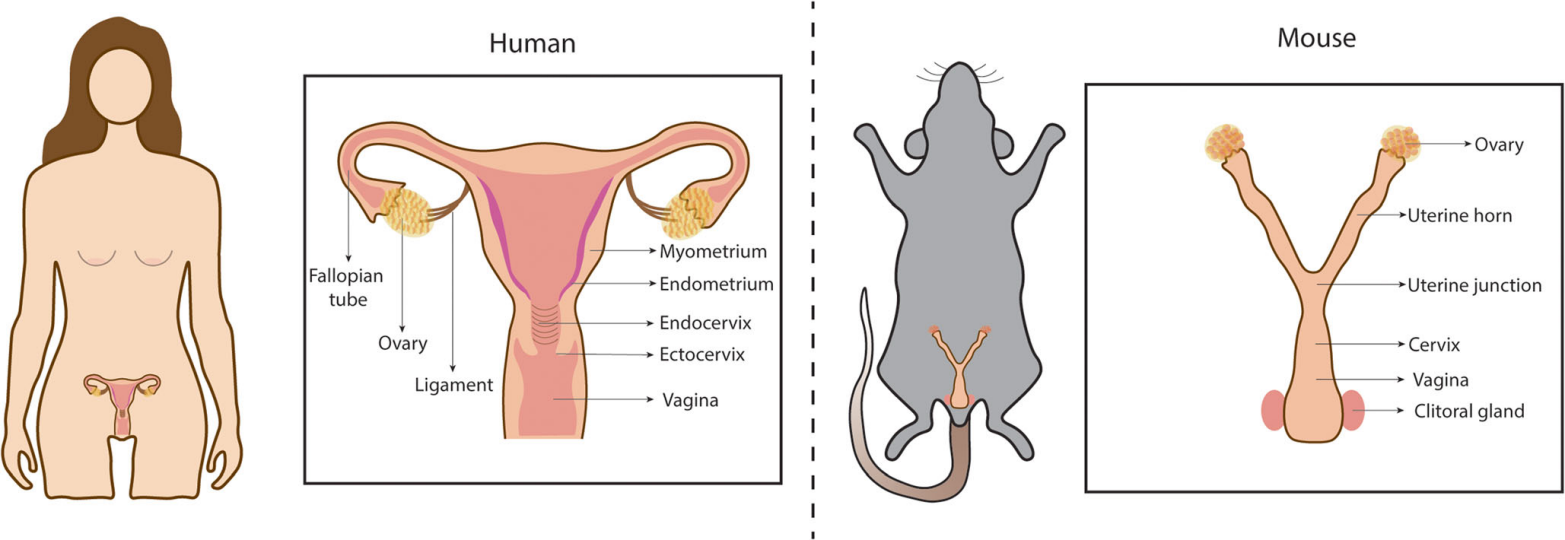
Anatomy of the human and mouse FRT. In humans, the FRT consists of ovaries, fallopian tubes, uterus (endometrium and myometrium), cervix (endocervix and ectocervix) and vagina. There are some anatomical differences between the two species, as mice have a bicornuate uterus (uterine horns) and oviducts are much less pronounced in proportion to the rest of the reproductive tract

The successful birth of healthy offspring, necessary for the continuation of the species, depends on the female reproductive tract (FRT). The FRT is responsible for regulating oocyte maturation, providing the protective environment for fertilisation and implantation of the embryo, as well as ensuring access to nutrition for fetal growth. This process concludes with parturition, and the FRT then undergoes remodelling to allow repetition of this remarkable process. Across allmammalian species there is a diverse range of reproductive strategies, for example, in the length and regulation of the estrous cycle, type of placentation and litter size, but the basic features are generally shared (Fig. 1) [1]. The FRT is composed of the ovaries, the site of maturation and release of oocytes; the fallopian tubes (FT, also called oviducts) that transport the oocytes to the uterus following ovulation; the uterus where implantation of the embryo occurs and pregnancy takes place; and the cervix which connects the uterus to the vagina and is the entry site for male gametes as well as the birth canal (Fig. 1). The cervix also acts as a barrier protecting the upper FRT from invading pathogens.

The regulation of FRT function is complex and is coordinated by hormones of the hypothalamus-pituitary-ovarian (HPO) axis [2]. The differential hormonal responses between the epithelial, stromal and immune populations provide another layer of regulation locally within the tissues. Disruption of these processes leads to a breakdown in homeostasis that may result not only reproductive failure but also in a range of disorders including endometriosis and carcinomas. These conditions affect large numbers of women. There is still limited understanding of their etiology and a lack of effective treatment options [3, 4]. Furthermore, increasing maternal age, diet and environmental factors (e.g. presence of endocrine-disrupting substances) and other changes in our lifestyle are contributing to a rise in the incidence of these disorders [5–7]. Thus, a better understanding of the cellular and molecular mechanisms controlling the function of the FRT is of increasing importance.

Although there have been recent, major advancements in reproductive research, key questions remain unresolved due to the complexity of the FRT and the lack of tractable experimental systems [8]. This is particularly true with regard to understanding the normal physiology and molecular regulation of the human FRT. Although different types of three-dimensional (3D) culture systems have been reported for the FRT, the long-term, stable expansion of these primary cells remains a challenge. The development of the 3D organoid culture system by Sato and Clevers that allows the culture and expansion of both normal and diseased cells in a defined medium has been a transformative tool for the study of many human tissues [9]. Organoids can be derived either from pluripotent stem cells (PSC) or from tissue-derived stem cells (fetal or adult). They are defined as cellular structures that self-organise, consist of multiple cell types and emulate key architectural, genetic and functional aspects of a specific tissue [10].

There are several differences between PSC-derived and tissue-derived organoids. PSC-derived organoids are generated by taking advantage of their inherent ability to undergo differentiation and spatial patterning to adopt specific cell fates in response to developmental cues [10]. Under specific culture conditions which include signals from the wingless-related integration site (Wnt), bone morphogenetic protein (BMP), epidermal growth factor (EGF) and fibroblast growth factor (FGF) pathways, PSC can be induced to undergo ‘organogenesis’ in a dish resulting in complex 3D structures that resemble organs containing multiple cell types. Many types of organoids have been derived from PSC, including intestine, liver, stomach, kidney, lung and brain [11–16].

Tissue-derived organoids are generated by embedding stem/progenitor cells into an extracellular matrix (ECM) (generally the commercially available Matrigel), which provides a basement membrane-like structure, and grown under conditions that mimic signals in the stem cell niche [9]. In contrast to PSC-derived organoids, those derived from tissues are only composed of epithelial cells. Tissue-derived organoids have been established from almost all major organs in humans and mice (reviewed in [10, 17]). Tissue-derived organoids can be propagated and expanded long-term under their optimal culture conditions (≥ 6 months) [18]. Although different types of organoids require a range of culture conditions, Wnt, EGF and BMP pathways are key. There are species-specific differences; some human organoids like those derived from the gut, pancreas and liver require additional factors to the corresponding mouse organoid media, including inhibition of transforming growth factor-beta (TGF-β) and p38 mitogen-activated protein kinase (MAPK) pathways, nicotinamide and forskolin [19, 20].

Recently, organoids have been generated from the murine and human FRT. In this review, we discuss these recent advancements with a focus on adult tissue-derived organoids and how they are shaping the way we can study both the normal physiology and disease of the FRT. We also highlight how this technology has the potential to improve women’s health.

## Anatomy and function of the adult human female reproductive tract

The key aspect of adult stem cell-derived 3D organoids is that they mimic the structure and function of the native adult tissue. Here, we introduce the basic anatomy, function and histology of the different parts of the FRT, with a focus on the mucosal layer and its epithelial cells.

### The ovaries

**Fig. 2:**
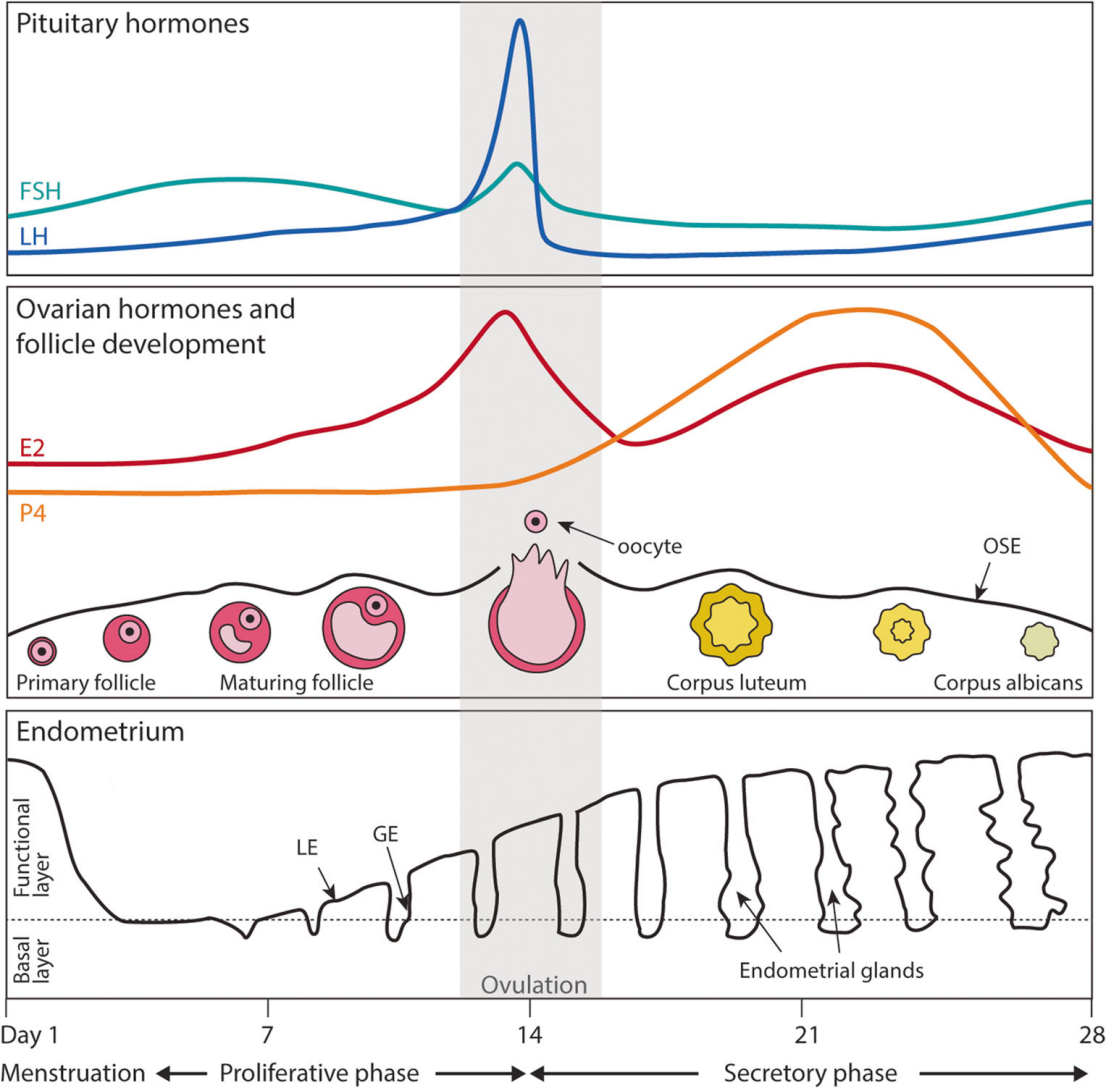
The menstrual cycle. The pituitary and ovarian hormones regulating the menstrual cycle and the morphological changes occurring at the ovarian surface epithelium (OSE) and endometrium are depicted. The menstrual phase is divided into menstrual, proliferative and secretory phases. During the proliferative phase, follicle-stimulating hormone (FSH) promotes the growth of ovarian follicles. This results in rising levels of oestrogen (E2) produced by the follicles which then drives the proliferation of the functional layer of the endometrium. Mid-cycle, a peak in E2 results in a surge of luteinizing hormone (LH) and the release of the oocyte (ovulation). This marks the start of the secretory phase, dominated by progesterone (P4), which drives the differentiation (decidualisation) of the endometrium to prepare for implantation. In the absence of implantation, P4 levels drop triggering menstruation, the shedding of the functional layer, to restart the cycle. The basal layer is not shed

The ovaries are found in close proximity to the fimbria of the FT and are kept in place by the ovarian ligaments attached to the uterus (Fig. 1). The ovaries have two functions: to produce and release mature oocytes into the FT and to act as an endocrine organ by secreting sex hormones oestrogen (E2) and progesterone (P4) (Fig. 2) [21]. E2 and P4 direct the cyclical changes of the mucosal lining of the uterus (endometrium) and thus play a vital role in fertility and health of the FRT. The ovaries are also the initial source of P4 once implantation occurs, which is essential to sustain pregnancy in the first few weeks [22, 23].

**Fig. 3:**
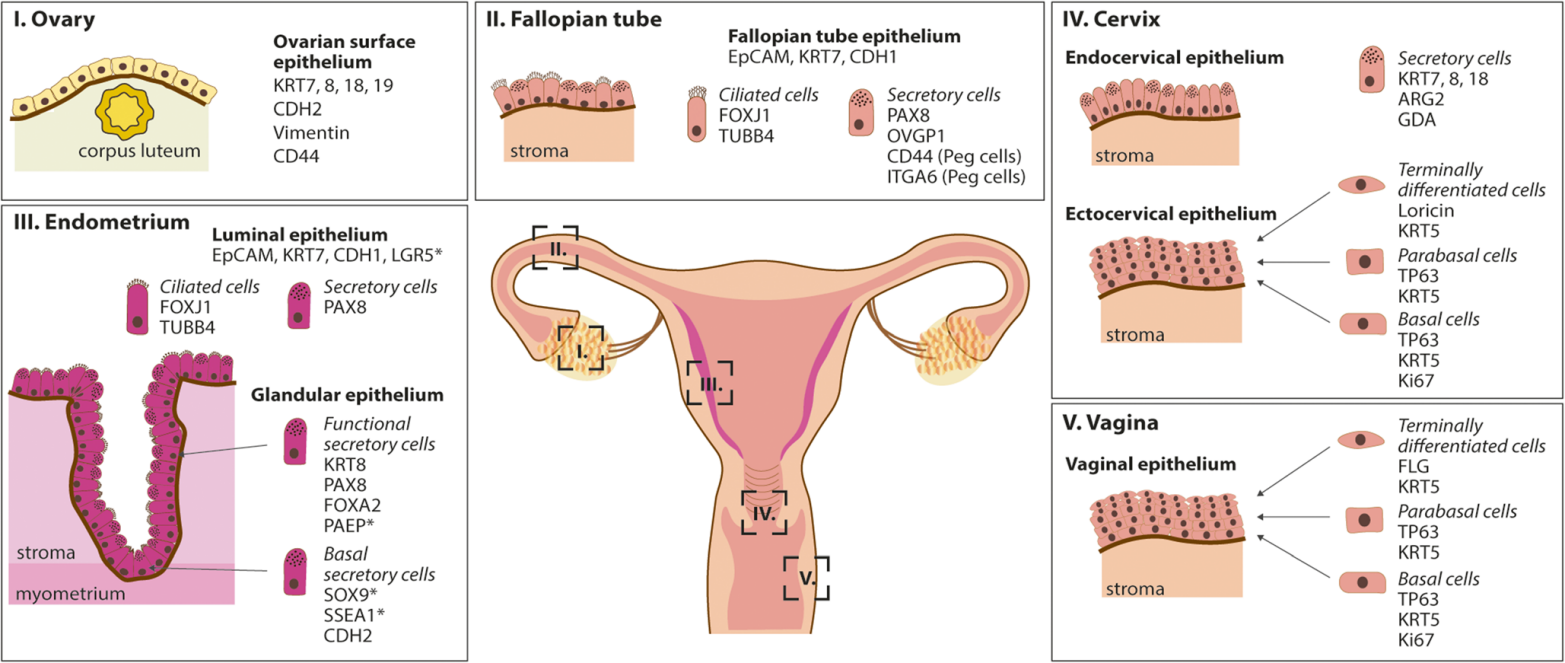
The epithelia of the human FRT. The types of epithelia covering the different regions of the human FRT are illustrated. For each tissue the major cell types are shown, with examples of typical markers they express. Markers with an asterisk are hormonally regulated

The ovaries are oval-shaped structures that can be divided histologically into the outer cortex and inner medulla. In most species, the cortex contains the follicles that are made up of oocytes and the supporting follicular cells. Blood vessels and lymphatics enter the ovary in the medulla. The HPO hormonal axis regulates ovarian function [24]. At the beginning of each menstrual cycle, several follicles begin to mature under the influence of follicle-stimulating hormone (FSH) released by the pituitary (Fig. 2). The follicles themselves begin to produce E2 and inhibin, inhibiting FSH production in a negative feedback loop. The follicles continue to mature and a peak in E2 production stimulates secretion of the luteinizing hormone (LH) from the pituitary gland. This results in ovulation where the follicle ruptures to release the oocyte from the ovarian surface epithelium (OSE). The OSE consists of a single layer of cuboidal epithelium, characterised by expression of epithelial markers, including cytokeratins KRT7, KRT8, KRT18 and KRT19, as well as mesenchymal markers CDH2 and vimentin (Fig. 3) [25]. Notably, unlike other simple epithelia, CDH1 is rarely observed in ovarian epithelium [26]. These molecular characteristics mean that OSE is plastic with the capacity to undergo bidirectional epithelial-mesenchymal conversion to allow repair of the ovarian surface after ovulation [27]. The lifelong OSE homeostasis and post-ovulatory repair in vivo are maintained by Leu-rich repeat-containing G protein-coupled receptor 5 (*Lgr5*)-expressing stem cells at the ovarian surface [28, 29]. Niche factors, including EGF and TGFβ signalling, regulate epithelial–mesenchymal transition (EMT) in OSE [29–31].

Upon ovulation, the oocyte is captured by the fimbria and transported into the FT where fertilisation takes place. The ruptured follicle transforms into the corpus luteum (CL) which secretes E2 and P4. If fertilisation and implantation occur, the CL continues to produce P4. In the absence of fertilisation, the CL degenerates, forming the corpus albicans, concomitantly resulting in a fall in P4 levels (Fig. 2). This triggers menstruation and the restart of the cycle. Dysregulation of these processes is responsible for many disorders which include ovarian cysts, ovarian carcinomas, disorders of the menstrual cycle and polycystic ovarian syndrome [32].

### The fallopian tubes

The FT are the link between the ovaries and the upper part of the uterus providing the passage for the oocytes released from the ovaries (Fig. 1). In humans, there are four sections of the FT: the infundibulum, a funnel-like structure in contact with the ovaries, the ampulla, the ampulla-isthmus junction and the isthmus. The surface epithelium of the FT contains secretory, ciliated and peg cells, and the segments differ in the proportion of these cell types and the complexity of the longitudinal folds present (Fig. 3) [33, 34]. The ciliated cells are particularly abundant in the infundibulum and ampulla [33]. They are essential for capture of the oocyte from the ovaries to guide it into the ampullary-isthmus junction where fertilisation takes place [35, 36]. Ciliated cells (acetylated α-tubulin+) are columnar and characterised by an oval nucleus with slender cilia protruding into the lumen. The secretory cells (PAX8+) are also columnar and contain apical secretory granules. Peg cells are found intercalated between ciliated and secretory cells; they have little cytoplasm and a dark staining nucleus [37]. All three cell types express epithelial cell adhesion molecule (EpCAM) whilst the peg cells also express CD44 and integrin α6 (ITGA6) (Fig. 3) [37, 38]. FT *Pax8*+ secretory cells act as stem cells and are enriched in the distal portion of the mouse FT including the fimbria [39–41]. Peg cells have secreted their products into the lumen and are an exhausted subpopulation of epithelial cells [42–45].

### The uterus

The uterus is composed of myometrium, a thick layer of smooth muscle, with an inner mucosal layer, the endometrium (Fig. 1). It provides the protective environment for the embryo to implant and to develop. The outermost layer in contact with the peritoneal cavity is the perimetrium. The myometrium undergoes contractions both in the non-pregnant state, during which it is thought to play a role in guiding the embryo before implantation, and in pregnancy to expel the baby at birth [46]. The endometrium is further divided into an upper functional layer facing the cavity of the uterus and the basal layer, juxtaposed to the myometrium (Figs. 2 and 3). The functional layer contains the surface luminal epithelium, upper portion of the glands whilst the basal layer contains the basal, lower portion of the glands.

The endometrium has an essential role in implantation and particularly during the early stages of pregnancy [47]. It is a regenerative, dynamic tissue as it undergoes dramatic changes in response to the ovarian hormones throughout the menstrual cycle (Fig. 2) [48]. The morphology, ultrastructure and biochemical characteristics of endometrial cells vary greatly throughout the cycle. Extending up from the myometrial margin, long tubular glands open at the surface of the endometrium that is covered by a luminal epithelium [49]. The luminal epithelium, like the FT, contains both acetylated α-tubulin+ ciliated cells and PAX8+ secretory cells (Fig. 3) [50]. The glandular epithelium is composed mostly of columnar secretory cells with a few ciliated cells at the gland openings [51]. Both the non-ciliated luminal and glandular epithelium are positive for EpCAM, CDH1 and KRT7 (Fig. 3) [50]. The stroma beneath the endometrial surface and between the endometrial glands contains CD10+, vimentin+ mesenchymal cells that undergo dramatic changes during pregnancy [52]. The blood is supplied from spiral arteries, and these play an important role in menstrual breakdown and in supplying nutrients to the fetus during pregnancy [53].

In humans, rising levels of E2 after menstruation mark the start of the proliferative phase during which the functional layer is regenerated and grows considerably (Fig. 2). It is thought to regenerate from the basal portion of the glands, which is not shed [49, 54–56]. The basal glands express markers found in other tissue stem cells/progenitors, including SRY-box transcription factor 9 (SOX9), stage-specific embryonic antigen 1 (SSEA1) and CDH2 (Fig. 3) [57–59]. This might be a stem cell compartment, supported by the observation that CDH2+ cells can form gland-like structures in vitro [57]. Recent studies have proposed the existence of different bipotent stem cells in the murine endometrium that are able to give rise to both luminal and glandular epithelium and are spatially restricted to either the intersectional zone (*Foxa2*+ cells) or basal region (*Axin2*+ cells) [60, 61]. The luminal epithelium may also be a source of stem cells/progenitors. *LGR5*, the receptor for Rspondin that potentiates Wnt signalling in stem cells of many tissues, is highly expressed in the luminal epithelium especially during the proliferative phase in humans (Fig. 3) [62]. In the adult mouse, *Lgr5* is also hormonally regulated but is restricted to the luminal epithelium during the diestrus phase [63]. However, long-term lineage tracing shows that they are not able to contribute to the different lineages, thus suggesting that *Lgr5*+ cells are rather short-lived differentiated cells of the adult endometrium in mice [63]. The murine endometrium does not undergo shedding and regeneration so it is likely that there are species-specific differences in how this tissue is regulated.

After ovulation, during the secretory phase, the increase in P4 levels triggers the process of decidualisation, which results in the specialised differentiation of the glands and stromal cells to prepare for pregnancy, accompanied by characteristic morphological and ultrastructural changes. The glands accumulate glycogen in the subnuclear cytoplasm, and they begin to secrete copious amounts of uterine milk proteins including glycodelin and osteopontin [64]. The stromal cells increase in volume and produce ECM proteins and play an essential role in the regulation of epithelial behaviour through their secretions that signal to the glands, including prolactin and insulin-like growth factor-binding proteins (IGFBP) [65–67]. In the absence of implantation, falling levels of P4 that result from the involuting CL trigger menstruation (Fig. 2). Decidualisation is essential for the establishment of pregnancy and defects in this process might contribute to several disorders of pregnancy (e.g. pre-eclampsia and miscarriage) [68–70].

### The cervix

The cervix links the uterine cavity with the vagina acting as a physical barrier between the external environment (vaginal canal) and the uterus (Fig. 1). The two major functions of the cervix are to facilitate the passage of spermatozoa into the uterine cavity and subsequently to FT where fertilisation takes place and to maintain sterility of the upper FRT [71, 72]. It consists of two regions, the ectocervix and the endocervical canal (endocervix). The endocervix is contiguous with the uterine cavity from which it is separated by the internal os. The ectocervix projects into the vagina. The external os marks the transition from the ectocervix to the endocervical canal. The columnar epithelium lining the endocervix meets the squamous epithelium of the ectocervix at the squamocolumnar junction (SCJ) [73]. The SCJ undergoes dynamic modification and under certain physiological or pathological conditions (influences of hormones especially in pregnancy, altered microbiota), the glandular epithelium at the SCJ is replaced by squamous metaplasia, creating a transition or transformation zone (TZ) [72, 74, 76].

The endocervical surface and gland-like crypts in the tissue are covered by columnar epithelium with basal nuclei. These epithelial cells express characteristic cytokeratins including KRT8, KRT7 and KRT18 and renew very slowly (Fig. 3) [77, 78]. Like the upper FRT, the columnar epithelium is composed of two major cell types, ciliated and secretory cells [76]. The secretory cells produce cervical mucus and the ciliated cells facilitate the movement of the mucus towards the vagina to prevent ascending infections and support the entrance of spermatozoa into the uterus [74]. The squamous epithelium of the ectocervix is composed of multiple layers: basal, parabasal, intermediate and superficial layers sitting on a basement membrane (Fig. 3). Cells move from the basal to superficial layers accompanied by cell differentiation and flattening (squamification) [79]. The cuboidal basal cells are progenitor cells that are essential for constant tissue regeneration and express the proliferation marker Ki67. The transit amplifying cells in the parabasal layer have limited proliferative capacity. Maturing squamous cells are present in the intermediate layer while the superficial layer consists of terminally differentiated non-keratinised flattened cells that are ready to exfoliate [79, 80]. The intermediate and superficial layers of the cervix and vagina epithelium are rich in glycogen which acts as a nutrient and supports the proliferation of healthy microbiota like *Lactobacillus* [81]. The squamous stratified epithelium is characterised by expression of cytokeratins including KRT5, KRT14 and KRT17, distinct from those of endocervix epithelium (Fig. 3) [78, 82]. Further, the transcription factor *Tp63*, a homologue of the tumour suppressor gene *Tp53*, is the master regulator and is essential for the development of squamous epithelium [75, 83].

Two distinct types of sub-epithelial stromal cell compartments underlie the ectocervical squamous and endocervical columnar epithelium [78, 84]. The ectocervical stroma is desmin+/smooth muscle actin (SMA)− whilst the endocervical stroma is desmin−/SMA+/CD34+ [84]. The regenerative potential of squamous and columnar epithelia is controlled by distinct Wnt signals from the stromal compartment. Endocervical stromal cells express Wnt signalling agonists, *AXIN2* and *RSPO1*, whilst the ectocervical stroma expresses a Wnt antagonist, Dickkopf-related protein 2 (*DKK2*), thus creating an opposing Wnt microenvironment at the SCJ [78]. The TZ is highly susceptible to persistent infections and is the most common site for the development of cervical neoplasia [85, 86]. Often squamous metaplasia precedes the development of the majority of cervical cancers [87, 88]. In a mouse model, a Wnt inhibitory environment promotes the emergence of foci of reserve cells that undergo squamous metaplasia; these are KRT5+ and are located under the columnar epithelium [78].

### The vagina

The vagina extends from the vulva to the uterine cervix (Fig. 1). It is distensible to allow for childbirth and is covered with rugal mucosal folds. The vagina prevents potentially invasive microorganisms from entering the uterus [89]. Healthy microbiota, including *Lactobacillus acidophilus* that colonise the vagina, secrete lactic acid, maintaining an acidic environment (pH 4.9–3.5) that reduces the chance of growth of pathogenic microorganisms [90–93]. However, dysbiosis, where disruptions in the healthy microbiome can allow even pathogenic members of the microbiome to take hold, results in a variety of infections with vaginitis being the most common [89, 94]. Despite the strong association with infection (e.g. human papillomavirus (HPV) infection), neoplasms are relatively unusual in this site, when compared with the development of carcinoma of the cervix [95].

Similar to the ectocervix, the mucosa of the vagina is lined with stratified squamous epithelium that is glycogenated and nonkeratinising. Vaginal regeneration is dependent on the basal cells that possess proliferative capacity and give rise to the TP63+, KRT17+, KRT5+ and KRT14+ basal progenitor cells and parabasal cells (Fig. 3). The differentiated intermediate layers express KRT13, CALML3 (calmodulin-like protein 3), KRT4 and IFITM3 (interferon-inducible transmembrane protein 3) and apical cornified terminally differentiated epithelium express KRT1 and KRT10 [96–98]**.**

## Organoids as a tool to study the female reproductive tract

**Fig. 4:**
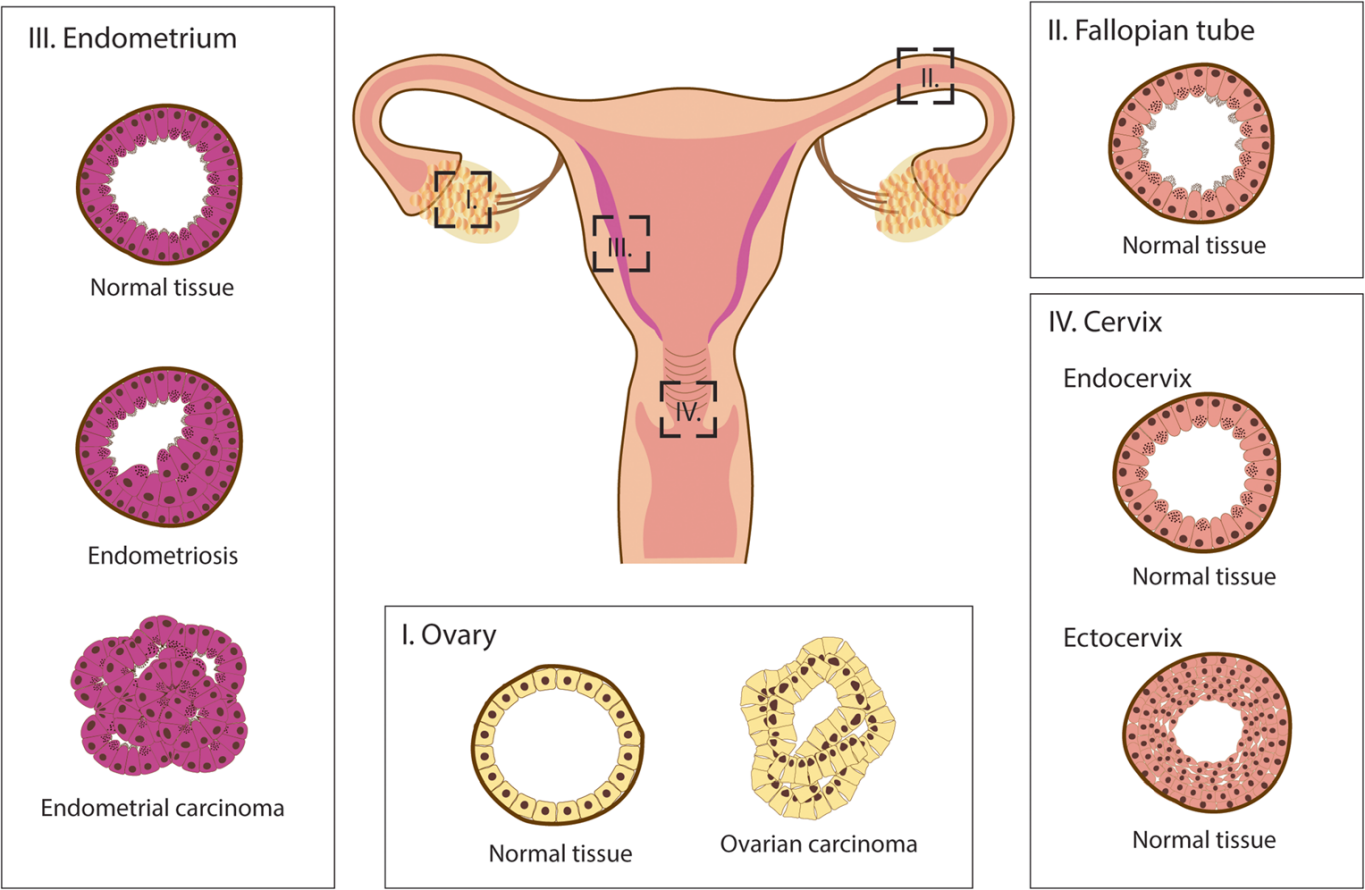
Organoids of normal and diseased tissues of the human FRT. Organoid models derived from normal and pathological tissues are illustrated. The different cell types that are present in the tissue epithelia and the organoids are shown as columnar (non-ciliated), secretory, ciliated, cuboidal and squamous. The organoids recapitulate cellular heterogeneity, genetic signature and key functions of the tissue of origin

Many in vitro and in vivo models are being used to study the biology and diseases of the FRT. Common in vitro models are primary cells isolated from tissues, cell lines established from carcinomas, tissue explants and 3D organotypic models [99–103]. Although these are important tools, there are several caveats. Primary cells have a limited life-span in culture and the cell lines commonly used, ECC-1 (endometrial carcinoma) and HeLA (cervical carcinoma), are karyotypically abnormal and do not represent the heterogeneity of the initial tumour mass due to selection for cells with proliferative capacity in vitro. Furthermore, many of the functions of the tissues are not fully recapitulated in monolayer cultures. On the other hand, although mouse models provide a much more physiologically relevant system, they are not cost effective and many of the human features are not reliably modelled due to considerable species-specific differences in functions of FRT. For example, the endometrium of the mouse does not undergo menstruation and spontaneous decidualisation [104]. 3D organoid cultures generated from the FRT, a recent advancement for this field, provide solutions to many of the limitations of the available model systems: they can be propagated long-term, function like the tissue of origin and are relatively cost-effective. Here, we summarise the recently established organoid systems of the FRT (Fig. 4).

### Fallopian tube and ovarian organoids

Various 3D models have been developed for the in vitro culture of primary human FT epithelium. For example, transwell based air-liquid interface cultures using a cell culture medium containing Dulbecco’s Modified Eagle’s Medium (DMEM)/Ham’s F12 1:1 supplemented with 2% serum substitute UltroserG were used to study FT biology and pathology. Although these cultures recapitulated some aspects of the in vivo architecture of the FT epithelium and consisted of polarised cells of both secretory and ciliated types, they cannot be propagated long-term [105, 106]. Subsequently, organoids that can be propagated long-term were derived from human FT that are EpCAM+ and contain both PAX8+ secretory and acetylated-α-tubulin+ ciliated cells (Fig. 4) [38]. Their long-term propagation is supported by growth factors that modulate Wnt, Notch, EGF, FGF and TGF-β signalling pathways (Table 1) [38]. Wnt and Notch are essential for the establishment of human FT organoids and inhibition of Notch promotes ciliary differentiation [38, 39]. Similarly, in the mouse oviduct, Wnt/β-catenin signalling is required for epithelial homeostasis and self-renewal of secretory cells [40]. Human FT organoids are hormonally responsive as the FT in vivo. Several genes that are thought to play an important role in fertilisation, including *PGR* and oviductal glycoprotein 1 (*OVGP1*), are inversely regulated by E2 and P4 during the menstrual cycle [38]. With hormonal treatment these were also regulated in the same way in FT organoids.

**Table 1:** Media components for the derivation and propagation of organoids from healthy and diseased human and mouse FRT. The growth factors and inhibitors required for the establishment of organoid cultures as well as their expansion for at least 10 passages are summarized. For each region of the FRT, organoids derived from healthy and pathological tissues are listed

FRT	Specie	Tissue source	Organoid medium^a^	Reference
Ovary	Human	Ovarian tissue from prophylactic bilateral salpingo-oophorectomy (with/without *BRCA1/BRCA*2 mutations)	Wnt3a CM, Rspondin-1 CM, Noggin CM, EGF, Nicotinamide, A83-01, Y-27632, NRG1, Forskolin, Hydrocortisone, β-Estradiol	[107]
	Human	Ovarian carcinoma (low and high grade serous adenocarcinoma, mucinous adenocarcinoma, clear cell carcinoma, endometrioid carcinoma, serous borderline tumour)	Wnt3a CM^b^, Rspondin-1 CM, Noggin CM, EGF, FGF10, Nicotinamide, A83-01, Y-27632, NRG1, Forskolin, Hydrocortisone, β-Estradiol	[107]
	Human	High-grade serous ovarian carcinoma (primary tumour deposits within abdominal cavity)	Wnt3a CM^c^, Rspondin-1 CM^c^, CHIR99021^c^, Noggin^c^, BMP2^c^, EGF, FGF10^c^, Nicotinamide, SB431542, Y-27632,	[108]
	Human	Ovarian carcinoma (low and high grade serous adenocarcinoma, mucinous adenocarcinoma, clear cell carcinoma)	Rspondin-1 rec/CM, Noggin rec/CM, EGF, FGF10^c^, FGF2^c^, IGF1^c^, HGF^c^, Nicotinamide, A83-01, Y-27632^d^, NRG1^c^, SB203580, β-Estradiol	[109]
	Mouse	Ovarian tissue	Wnt3a CM, Rspondin-1 CM, Noggin, EGF, Nicotinamide, A83-01, Hydrocortisone, β-Estradiol	[110]
Fallopian tube	Human	Anatomically normal FT tissue (removed for benign gynaecological diseases)	Wnt3a CM, Rspondin-1 CM, Noggin, Nicotinamide, EGF, FGF10, Y-27632, SB431542	[38]
	Human	FT tissue from prophylactic bilateral salpingo-oophorectomy (with/without *BRCA1/BRCA*2 mutations)	Wnt3a CM, Rspondin-1 CM, Noggin CM, Nicotinamide, EGF, A83-01, Y-27632	[107]
	Mouse	FT fimbriae	Wnt3a CM, Rspondin-1 rec/CM, Noggin, EGF, Y-27632^e^	[110]
	Mouse	FT tissue	Wnt3a^f^, Rspondin-1^f^, Noggin^f^, EGF, FGF10^f^, SB431542, Y-27632	[39]
Endometrium	Human	Endometrial biopsies (proliferative, secretory and postmenopausal phases); decidual tissue (first trimester termination samples, 6–9 weeks gestation from normal pregnancy)	Rspondin-1, Noggin, EGF, FGF10, HGF, Nicotinamide, A83-01, Y-27632^g^	[50]
	Human	Endometrial biopsies in proliferative phase (laparoscopy for benign gynaecological conditions)	Wnt3a CM, Rspondin-1 CM, Noggin, EGF, FGF10, Nicotinamide, A83-01, Y-27632^d^, SB202190, β-Estradiol	[111]
	Human	Decidua (first trimester termination samples, 8–11 weeks gestation from normal pregnancy)	Rspondin-1, CHIR99021, Noggin, EGF, A83-01, PGE2	[112]
	Human	Endometrial tissue (menstrual, proliferative, secretory phases)	Rspondin-1 CM, Noggin rec/CM, EGF, FGF2, FGF10, Nicotinamide, A83-01, Y-276322^d^, SB202190, β-Estradiol	[113]
	Human	Ectopic (stages I–IV) and eutopic endometrium (proliferative, secretory phases) from endometriosis	Rspondin-1 CM, Noggin rec/CM, EGF, FGF2, FGF10, Nicotinamide, A83-01, Y-276322^c^, SB202190, β-Estradiol	[113]
	Human	Endometrial hyperplasia (simple benign, complex atypia, Lynch syndrome)	Rspondin-1 CM, Noggin rec/CM, EGF, FGF2, FGF10, Nicotinamide, A83-01, Y-276322^d^, SB202190, β-Estradiol	[113]
	Human	Endometrioid, clear cell, serous endometrial carcinoma (grades I–III)	Rspondin-1 CM, Noggin rec/CM, EGF, IGF1, HGF, Nicotinamide, A83-01, Y-27632^d^, SB202190, β Estradiol	[113]
	Mouse	Uterine horns (estrous phase)	Wnt3a CM, Rspondin-1 CM, Noggin, EGF, FGF10, Nicotinamide, A83-01, Y-27632^d^	[111]
Cervix	Human	Anatomically normal endocervical biopsies	Wnt3a CM, Rspondin-1 CM, Noggin, EGF, FGF10, Nicotinamide, SB431542, Y-27632	[78]
	Human	Anatomically normal ectocervical biopsies	Noggin, EGF, FGF10, Nicotinamide, SB431542, Y-27632, Forskolin, Hydrocortisone	[78]
	Mouse	Endocervical tissue	Wnt3a CM, Rspondin-1 CM, Noggin, EGF, FGF10, Nicotinamide, Y-27633, SB431542	[78]
	Mouse	Ectocervical tissue	Noggin, EGF, FGF10, Nicotinamide, SB431542, Y-27632	[78]
Vagina	Mouse	Vaginal tissue	EGF, A83-01, Y-27632, Ultraserum-G	[96]

*FT* fallopian tube, *CM* conditioned medium, *rec* recombinant, *EGF* epidermal growth factor, *FGF* fibroblast growth factor, *HGF* hepatocyte growth factor, *A83-01* TGFβ receptor inhibitor, *NRG1* neuregulin 1, *SB431542* TGFβ receptor inhibitor, *SB202190* p38 MAPK inhibitor, *SB203580* p38 MAPK inhibitor, *Y-27632* ROCK inhibitor, *CHIR99021* GSK-3 inhibitor, *PGE2* prostaglandin E2

^a^Excluding basal medium components (i.e. advanced DMEM/F12, N2, B27, insulin-transferrin-selenium (ITS), Glutamax, N-acetylcysteine, antibiotics, HEPES); all factors are recombinant if not designated ‘CM’

^b^Some tumour organoids require Wnt3a CM

^c^Supplementation/omission of these factors depends on the sample

^d^Supplementation only for initiation of organoid cultures or after passaging

^e^Supplement for recovery of organoids after thawing

^f^Not essential for long-term culture but results in more mature organoids

^g^Supplement for derivation and expansion of clonal organoid cultures or for recovery of organoids after thawing

Human OSE has been difficult to grow and many attempts have been made to establish long-term cultures. In an initial report of 3D cultures of human OSE, primary cells isolated from normal ovaries were resuspended in a medium containing 10% serum and cultured on a Matrigel coated well, which resulted in the formation of spheroids that were positive for OSE markers including calerectin and cytokeratins (stained with antibody cocktail KRTAE1) [114]. However, these are not long-term cultures. A recent study has established definitive organoids of human OSE (Fig. 4) [107]. FT and OSE from women with a high risk of ovarian cancer due to germline mutations in *BRCA1/2* genes were used to derive organoids (Table 1) [107]. OSE organoid lines were established with > 90% efficiency but they grew more slowly than the FT-derived organoids. OSE organoids are KRT8+ and show typical folds and invaginations similar to FT organoids. However, further work is needed to improve their ability to be propagated long-term. Organoids derived from murine OSE can be passaged indefinitely under culture conditions containing Wnt3a, Rspondin-1, Noggin, EGF, nicotinamide, hydrocortisone and β-estradiol (Table 1) [110]. Similarly, to the human FT organoids, these are cystic with mucosal folds and contain both ciliated and secretory cells. These models show great promise for the study of FT and OSE biology.

### Endometrial organoids

The ability of endometrial epithelium to form 3D structures in vitro was first described using primary cell isolates grown as a monolayer on Matrigel in serum-free conditions that spontaneously formed 3D glandular-like structures [115]. These tubular structures contain a central lumen lined by cells with microvilli. Subsequently, spheroidal cultures of murine endometrial epithelium with polarised, CDH1+ cells surrounding a central lumen were generated by culturing them in a medium containing EGF, insulin-transferrin-selenium supplements and Matrigel [116, 117]. A similar spheroidal culture method was used to investigate the stem cell/progenitor characteristics of SSEA1+ and CDH2+ human endometrial epithelial cells located in the basal layer, in two different studies [57, 59]. Both SSEA1+ and CDH2+ cells are capable of forming spheroidal, gland-like structures at higher efficiency than their negative counterparts. Furthermore, SSEA1+ cells from women with endometriosis, a disorder where endometrial epithelial and stromal cells grow outside the uterine cavity, also showed higher spheroid forming capacity compared with normal endometria, providing further evidence for their stem cell ability [58]. The spheroid assays are a useful method to investigate proliferative and clonal capacities; however, the long-term ability of the cultures has not been shown.

Using the approach established by the Clevers lab, endometrial organoids (EO) that can be expanded and cultured long-term were established using endometrial tissue from both human and mice (Fig. 4) [50, 111]. Human EO can be derived from all stages of the menstrual cycle, including from menopausal and pregnant endometrial tissues (decidua) with near 100% success rate, whilst in mice, establishment of EO is most efficient when samples are obtained at the estrous phase [50, 111]. EO from both species have similar morphology and grow as spheroids with the apical surface of the cells lining the central lumen. Similar conditions used in other organoids systems are needed to grow EO. Murine EO are grown in Wnt3a and Rspondin-1 conditioned medium supplemented with EGF, FGF-10, Noggin, nicotinamide and TGF-β inhibitor and can be expanded every 7–10 days for several months (Table 1) [111]. Human EO grow in similar conditions but addition of exogenous Wnt is not necessary [50]. There are different human EO media with the addition or omission of factors including hepatocyte growth factor (HGF), E2, p38 MAPK inhibitor, prostaglandin E2 (PGE2), β-estradiol and Wnt activator (CHIR99021) (Table 1) [50, 111, 112]. EO can be cultured extensively in these media variations but it remains to be investigated whether the cellular composition and cell states of the organoids are different.

EO faithfully recapitulate the morphological and molecular features of endometrial glands in vivo. A comparison by microarray of original glandular fragments with organoids from the same patient sample shows high similarity in their gene expression profiles; for example, genes of the secretory lineage and of murine endometrial glands, *PAX8*, *SOX17* and *FOXA2*, are uniformly expressed in human EO [50]. EO can functionally respond to ovarian hormones E2 and P4, similar to in vivo epithelium by producing glandular milk proteins, osteopontin and glycodelin, and forming ciliated cells [50]. They also have clonogenic ability as organoids can be generated from a small proportion of cells (between 1 and 3%) by limiting dilution assay [50]. These clonal cultures are bipotential and can differentiate into both secretory and ciliated cells providing an opportunity to study the cell lineage relationships and the regenerative ability of endometrial epithelial cells. Concomitant NOTCH inhibition and E2 signalling in directing the differentiation towards the ciliated cells were demonstrated using EO [112]. Functional studies using EO have also revealed a potential role of the mechanosensitive ion channel, PIEZO1, in promoting implantation [118].

A major goal in the field is to identify stem cells of the endometrium. In mice, *Axin2*+ cells that reside in the basal glands are able to give rise to fully functional endometrial organoids that can generate both ciliated and glandular lineages [60]. The identity of endometrial stem/progenitor cells in the human endometrium remains to be established but this work paves the way to investigate whether a similar population exists using human EO. Furthermore, applications of recent technologies including single-cell RNA-sequencing and spatial transcriptomics will allow the investigation of potential markers to isolate and localise these cells in vivo [119, 120,121, 122].

### Cervical organoids

Recently, long-term, expandable organoid models of the squamous stratified ectocervix and columnar endocervix have been developed (Fig. 4) [78]. In line with the distinct molecular microenvironment that underlies these two epithelial types in vivo, the ecto- and endocervical organoids also depend on distinct growth factor combinations for their formation and long-term expansion [78]. In vitro recapitulation of organoids from human and mouse ectocervix depends on EGF, FGF-10, hydrocortisone, active BMP signalling and inhibition of TGF-β and ROCK (Table 1). Presence of Wnt signalling agonists (Wnt3a and Rspondin-1) are inhibitory for the development of human ectocervical organoids [78]. Additionally, forskolin, an inducer of cyclic adenosine 3′,5′-cyclic monophosphate (AMP), further supports the long-term propagation of these organoids. These organoids from mouse and human are similar and mimic the in vivo ectocervical epithelial tissue architecture [78]. They consist of multilayered KRT5+ epithelia with basal proliferative TP63+ cells superimposed by parabasal layers that have low TP63 expression and with more differentiated cells towards the inner lumen [78]. Differentiation of ectocervical organoids is dependent on Notch signalling as its inhibition leads to a failure in stratification, resulting in a TP63+ monolayer [78]. Using similar growth conditions, primary ectocervical epithelium can be grown in a transwell based air-liquid interface culture system so that the differentiated epithelial layers are amenable to pathogens or drug treatments, mimicking the in vivo situation [123].

Endocervical organoids have been generated from human and mouse endocervical tissue [78]. Maintenance of these cultures requires Wnt3a and Rspondin-1, EGF, FGF-10, active BMP signalling and inhibitors of TGF-β and ROCK (Table 1) [78]. They are composed of a monolayer of columnar, polarised cells with sporadically proliferating cells recapitulating the in vivo architecture of the endocervix [78]. Molecular characteristics include expression of KRT8, KRT7, KRT18, arginase 2 (ARG2) and guanine deaminase (GDA) [78]. Based on cytokeratin expression profiles visualised by immunohistology, small cuboidal subcolumnar reserve cells in the TZ and endocervix are the likely multipotent progenitors that can regenerate both columnar and squamous stratified cervical epithelia. By using murine organoids and lineage tracing in vivo, it is clear that the ecto- (KRT5+) and endocervical (KRT8+) epithelia are derived from two distinct lineages that merge at SCJ [78]. Furthermore, in agreement with these studies, in the absence of Wnt3a and Rspondin-1, human organoid models recapitulate the development of squamous metaplasia from the endocervical KRT5+ reserve cells that are distinct from KRT8+ glandular cell lineage [78]. Thus, cervical organoids are surrogate models for understanding cervical homeostasis, as their responses, for instance to Wnt growth factors, recapitulate the dynamics of metaplasia at the SCJ in vivo.

### Vaginal organoids

The culture and long-term maintenance of murine vaginal organoids require 2% Ultraserum-G, EGF, TGF-β and ROCK inhibitors (Table 1) [96]. Human vaginal organoids have not been described yet. Organoids from mice have a stratified squamous epithelium with TP63+ cells at the periphery, mimicking the architecture of vaginal tissue in vivo. Vaginal organoids depend on cell-autonomous Wnt signalling for their proliferation and differentiation [96]. Treatment with IWP2, a small-molecule inhibitor of Wnt signalling, or conditional ablation of β-catenin, resulted in decreased size and formation of organoids [96]. Although increasing Wnt in the medium improved the growth of organoids, high concentrations had the opposite effect, suggesting that fine regulation of Wnt activation regulates vaginal epithelial behaviour. This model will be essential for the study of the mechanisms underlying the regeneration and homeostasis of the vaginal epithelium.

## Organoid systems to model diseases of the female reproductive tract

With the recent surge of reports of organoids from the FRT, studies are appearing describing their use in modelling a range of pathological conditions. Here, we review the diseases of the FRT that have been modelled to date (Fig. 4).

### Endometriosis

Endometriosis occurs when endometrial tissue is found outside the mucosal lining of the uterine cavity. These endometriotic lesions are found in several sites including the ovaries, pelvic peritoneum and small and large bowel. Endometriosis affects ~ 10% of women of reproductive age [124]. A genetic contribution is likely but the etiology, pathogenesis and underlying biology of endometriosis remain essentially unknown [125–128]. Several sources of endometriotic lesions have been suggested including retrograde menstruation of endometrial stem/progenitor populations, metaplasia of peritoneal mesothelium and lymphatic and vascular metastasis of endometrial cells [129–132]. The ectopic endometrial lesions contain glands, stroma as well as uterine natural killer cells. They are E2 responsive and invasive, causing debilitating pelvic pain and chronic inflammation [128]. Depending on the number, depth and location of lesions, endometriosis can be staged from grade I (minimal) to grade IV (severe) [133, 134].

Medical management consists in reducing E2 production by administration of oral contraceptives, progestogens, GnRH agonists or androgen steroids [135]. However, hormonal treatment is only suitable for women who are not planning to conceive and there are considerable side effects. If medical intervention is unsuccessful, removal of the endometriotic lesions by surgery is an option but success depends on the number, location and stage of diseases and recurrence occurs in 30–50% of women [136, 137]. Hysterectomy and/or oophorectomy reduces recurrence but this may not be an option in women of reproductive age. Endometriosis is not only a huge burden on the quality of life, it is also associated with infertility as well as higher susceptibility to ovarian and breast cancer [138].

A recent study holds great promise for the study of endometriosis. A biobank of EO was established from endometriotic lesions from different clinical stages, including paired organoids from ectopic (ECT) and eutopic endometrium (EUT) from the same patient (*N* = 15) [113]. These organoids are grown in a medium based on their previous endometrial organoid medium but with addition of FGF2 and omission of Wnt3a (Table 1) [111, 113]. The morphology of the ECT organoid differed from EO and EUT organoids as they show hyperplastic epithelium (Fig. 4). ECT organoids maintain expression of epithelial markers CDH1 and cytokeratin as well as hormone receptors, E2 receptor α (ESR1) and P4 receptor (PGR). Despite the variability of gene expression profiles within the ECT organoid group, the RNA sequencing comparison to EO revealed alterations in PI3K-AKT, Wnt and hormonal responsive signalling pathways. Furthermore, characteristic known transcripts of endometriosis (aberrant expression of matrix metalloproteinase (MMP), integrins and the production of inflammatory cytokines IL-1β and IL-8) were also higher in ECT organoids [113, 139–141]. The inherent ability of human ECT organoids to establish and form ectopic endometriotic lesions was demonstrated by injection into murine intraperitoneal cavities [113]. However, it is important to note that stromal and immune populations, which are essential components of endometriotic lesions, are not recapitulated in this model. The organoid model of endometriosis recapitulates key epithelial features of the disease and will allow investigation of signalling pathways and genes that may drive the pathology, essential information for the discovery of new non-hormonal therapies.

### Endometrial carcinoma

Endometrial carcinoma (EC) is the most common gynaecological malignancy, the fifth most common cancer in women, and has an increasing prevalence [142]. Risk factors include endogenous and exogenous exposure to oestrogens, use of tamoxifen, nulliparity, early-onset menarche and late-onset menopause. Traditionally, EC has been classified into two main subtypes based on histological characteristics, hormone receptor expression and grade: endometrioid (type I) and serous (type II) tumours [143]. Type I is correlated with excess oestrogen, obesity, diploid tumour cells, hormone receptor positivity and a good outcome. Type II is common in older women and has a poor prognosis; there are abundant aneuploid cells and no expression of hormone receptors. The most frequent alterations in type I EC are: mutations in the phosphatidylinositol-4,5-bisphosphate 3-kinase (PIK3CA) pathway, with 90% of tumours showing loss of phosphatase and tensin homologue (*PTEN*) [144]; mutations in KRAS found in 20% of tumours [145]; mutations in fibroblast growth factor receptor 2 (*FGFR2*) in 12% of tumours [146]; amplification of human epidermal growth factor receptor 2 **(***HER-2*) in some subtypes [147]; loss of DNA mismatch repair (MMR) proteins (found in hereditary Lynch syndrome) [148]. Type II is more diverse with a range of histological subtypes; some tumours show features of serous ovarian carcinoma [149]. Thus, it is increasingly clear that when the pathology is combined with molecular and genetic features, there is a range of distinct EC subtypes [150, 151].

To investigate the cell-of-origin of endometrial hyperplasia and adenocarcinomas, two pathways commonly perturbed in human EC (PI3K and Wnt) were introduced into a mouse model that allows the tracing of *Axin2*+ stem cells [60]. After tamoxifen induction (which mimics exposure to oestrogen), carcinomas were indeed found throughout the uterus. Furthermore, these were a result of clonal expansion of mutant *Axin2+* cells, suggesting that upon oncogenic transformation, *Axin2*+ cells may be responsible for the development of EC. Indeed, the enhanced growth capacity of these cells was demonstrated by their ability to generate higher numbers of organoids than wild type, and they presented typical features of tumouroids with branched morphology and loss of the lumen [60]. Furthermore, when transplanted into chicken embryo chorionic allantois membranes, mutant *Axin2*+ cells were able to form organoids even without the presence of niche factors Wnt and Rspondin, in contrast to wild-type cells. *AXIN2* is also expressed in human endometrial glands [60]. Studies have revealed the clonal nature of endometrial glands in humans which frequently harbour driver mutations in cancer genes [152–154]. A similar forward genetic approach using human endometrial organoids may help elucidate the etiology of EC.

Several studies have reported the derivation of organoids from EC (Fig. 4) [50, 113, 155]. In the first proof-of-principle experiment, organoids were derived from endometrial adenocarcinomas, together with the adjacent normal endometrium using the same expansion medium for EO (Table 1) [50]. The architecture of the tumour organoids resembles the primary tumour; there is a multi-layered epithelium instead of the normal simple columnar epithelium, mitoses are frequent and isolated cells are present in the surrounding Matrigel. EC organoids were then generated from hysterectomy specimens from type I EC at different stages (*N* = 15) [155]. The dissociated malignant cells were embedded into basement membrane extract (BME) and grown in organoid medium containing nicotinamide, TGF-β inhibitor, p38 MAPK inhibitor, Rho kinase inhibitor and 17-β estradiol loosely based on organoid medium used for colonic carcinoma organoids [156]. The addition of the more commonly used organoid growth factors, EGF, FGF2, FGF-10, PGE2, Noggin, Rspondin-1 and Wnt3a, was not required for derivation although they might be needed for long-term growth. Morphological and histological characteristics like the expression of ESR1 and PGR reflected those of the original tumours. Several drugs were tested at the derivation stage to monitor effects on organoid establishment. This resulted in the identification of a new therapeutic STAT3 inhibitor, which showed potent growth inhibition of established 10 EC organoid lines [155].

The remarkable ability of the organoid method to generate cellular structures that faithfully recapitulate the genomic and transcriptomic features of the tumour is demonstrated in a recent study in which EO were derived from different stages of EC (*N* = 30), including endometrial hyperplasia, the pre-malignant state of EC [113]. Organoids were generated from different types of endometrial hyperplasia (benign, complex atypia and polyp) at 70% efficiency and could be grown long-term. These organoids showed a disorganised epithelium, though a central lumen was present. Specific genetic alterations found in the primary lesions, such as absence of TP53 and mutations in the MMR genes from the Lynch syndrome patients, were maintained in these organoids [113]. The derivation efficiency of EC organoids was much lower (20%) and they showed limited ability to propagate, with non-cancerous endometrial organoids overtaking the cultures. However, modification of their organoid medium with the removal of p38 MAPK inhibitor and addition of HGF, IGF1 and lipids enhanced tumour organoid formation, improved their ability to propagate and increased clonogenic ability (Table 1). The organoid lines had different morphologies with high-grade tumours displaying more dense structures with a lack of lumen. The EC organoids captured both the genetic, histological features and transcriptional similarity to the tumour of origin [113]. For example, using comparative genomic hybridisation or low-coverage whole-genome sequencing, the organoids were shown to retain a large portion of somatic copy number aberration (SCNA) found in the tumours, even with long-term passaging. A comparative transcriptomic analysis between EO, organoids derived from endometrial hyperplasia and EC showed over representation of PI3K-AKT signalling pathway as well as higher expression of genes involved in epithelial-mesenchymal transition [113].

Drugs targeting the molecular and genetic pathways altered in individual tumours have not yet been introduced on a routine basis and current treatment is total hysterectomy and use of adjuvant treatment (radiotherapy or chemotherapy) in women with extra-uterine or high-risk tumours. Identification of driver mutations within tumours that can be targeted specifically, for example trastuzumab for the treatment of breast cancer with amplification of the gene encoding epidermal growth factor receptor *HER2*, could be the way forward for EC [157]. However, the use of gene-drug associations to identify therapeutic treatments is not always informative as the actual number of targetable mutations that result in an effective therapy is relatively low [158]. Thus, to have a platform to screen for differential drug sensitivity and identify an effective treatment of EC would be of great benefit. Using a biobank of EC organoids, a small drug screen has shown the potential of this model for patient-specific targeted therapy. Five different EC organoid lines (derived from grades I to III) were grown in the presence of several chemotherapeutic agents (5FU, carboplatin, doxorubicin, everolimus, paclitaxel) and their viability was measured using the XTT (tetrazolium dye) cell proliferation assay [113]. The organoids showed different responses to the drugs, showing promise for personalised medicine approaches.

### Ovarian carcinoma

Ovarian carcinoma (OC) is a heterogeneous disease encompassing a wide diversity of tumour subtypes with distinct genetic and pathological characteristics. It affects ~ 225,500 women worldwide every year and is the 7th most common cancer-related death in women [142]. Risk factors include genetic factors, age and infertility [159]. The late diagnosis, aggressive nature and lack of effective treatment and screening strategies are responsible for high mortality rates with 5-year overall survival at 25% for patients with advanced stages of the disease [159]. OC can be categorised into three subgroups: border-line tumours, type I which includes low-grade serous carcinoma, clear-cell carcinoma, mucinous carcinoma and low-grade endometrioid carcinoma and type II high-grade serous carcinoma (HGSC) and high-grade endometrioid carcinoma [159, 160]. Although different mutations have been associated with some OC subtypes, the molecular mechanisms driving the pathogenesis remains unknown.

Given their ability to represent the heterogeneity of the tumour, the OC organoids hold great promise to study the biology and pathogenesis in order to improve clinical management and treatment of patients. In the first report, organoids were derived from HGSC, the highest grade and most common form of ovarian cancer (Fig. 4) [161]. Primary, metastatic or recurrent tumours were enzymatically digested and grown in Matrigel using a medium containing Rspondin-1, Noggin, EGF, FGF2 and 10, nicotinamide, PGE2 and inhibitors of TGF-β and p38 MAPK signalling (Table 1). The organoids showed key morphological features of HGSC like nuclear pleomorphism and disorganised epithelium but could only be expanded short-term. A more recent study reported further improvement of the HGSC organoid system and 15 organoid lines were established with 30% efficiency from advanced HGSC deposits [108]. HGSC organoids require a substantially modified growth medium in keeping with the modification of the niche factors seen in other tumour organoids [162]. EGF was the only indispensable component and Wnt activation was detrimental for HGSC organoid growth [108]. This optimised OC medium that consists of EGF, BMP2, nicotinamide and inhibitors of ROCK and TGF-β allows long-term culture of HGSC organoids that maintain key histological and morphological characteristics of the tumours of origin (Table 1) [108]. Organoids were also derived from the whole spectrum of OC subtypes and additional compounds during organoid derivation identified hydrocortisone, forskolin and NRG1 as beneficial, which was also confirmed by another study (Table 1) [107, 109]. Fifty-six organoid lines were derived at 65% efficiency; 85% of the lines recovered after cryopreservation, a prerequisite for the creation of a bio-bank [107].

The diverse morphologies characteristic of OC are typical of the range of organoid structures seen. There is a close correlation between the histological and molecular features of the organoids with the original tumour samples [107–109]. Thus, organoids of ovarian serous carcinomas are PAX8+ with a similar pattern of TP53 expression. Somatic mutations of *BRAF* and *KRAS* are frequent in some tumour subtypes and are retained in the corresponding organoids even after long-term passaging. The same is true for global epigenetic profiles of DNA methylation. Transcriptomic profiles of 35 OC organoid lines cluster with the original tumour samples and not with unrelated organoids [107]. Furthermore, this analysis revealed interesting biological insights into similarities between tumour types. The authors found that one type of pre-malignant lesion clusters with a specific subtype of OC, shedding light on precursors of invasive carcinomas. Thus, these models present an opportunity to study the pathogenesis of OC.

The great diversity of types of OC means that drug sensitivity tests are particularly valuable for these tumours. Twenty-one HGSC and non-HGSC organoid lines were treated with a panel of drugs and responses were measured by dose-response curves based on viability [107]. The majority of organoid lines responded to known treatments. For example, the genetic features of HGSC render the tumours sensitive to DNA damage, and in fact, a large proportion of HGSC organoids were sensitive to carboplatin/paclitaxel drugs whilst others were less so. This means new treatments can be tested on these non-responsive tumours in vitro like non-cisplatin compounds, including inhibitors of the PI3K/AKT/mTOR pathway, Poly(ADP-Ribose) Polymerase 1 (PARP), the tyrosine kinase, Wee1 and gemcitabine. There are differences in drug sensitivity even within the same tumour type and organoids derived from recurrent chemoresistant tumours. To provide proof that this drug sensitivity is maintained in vivo, when HGSC organoids sensitive to gemcitabine, a nucleoside analogue, were transplanted into mice, treatment with gemcitabine reduced tumour growth [107]. Thus, the OC organoids would allow pre-clinical functional assessment of the tumour to the drug.

Studies using mouse models and single-cell analysis of human fallopian tube cells suggest that HGSC can originate from the fallopian tube epithelium [41, 44, 163–166]. However, distinct subtypes of HSGC can also originate from the ovarian surface epithelium [110]. Recently, three commonly mutated genes *TP53*, *RB1* and *PTEN* found in HGSC were knocked down by short hairpin RNA (shRNA) in human FT organoids to model the FT-derived HGSC development [108]. Several changes suggest pro-carcinogenic progression in these organoids including apical-basal polarity and functional responses to the altered targeted loci, for example the resistance to apoptosis by Nutlin-3A, due to loss of *TP53*. However, they can still undergo differentiation to ciliated cells indicating that they have not fully transformed. When grown in the optimised ovarian carcinoma medium, stem cell markers, *CD133* and *SOX2*, increase and differentiation genes, *PGR* and *FOXJ1*, decrease suggesting that in the context of these genetic changes, the absence of Wnt maintains their altered proliferative state.

### Cervical carcinoma

Carcinomas of the cervix are the leading gynaecological cancer of women with more than half a million cases diagnosed and 300,000 deaths worldwide each year [142]. Cervical carcinoma occurs as two major, histologically distinct types: adenocarcinomas (ADC) and squamous cell carcinomas (SCC), constituting 9% and 90% of all the cervical cancers, respectively. Failure of early detection is the main cause of death; otherwise, the disease is treatable with surgery or chemo-radiation, or a combination of both. Persistent HPV infections with particular HPV subtypes are the etiological agent in virtually all cases. Prophylactic vaccines against these carcinogenic HPV types are available; however, the number of people receiving these remains low and new therapeutic approaches are needed [167].

The spectrum of histopathological changes seen in HPV infection that leads to cervical carcinoma reveals how the disease develops. There is dysplasia of squamous epithelium and the proportion of these cells present determines the grade of the dysplasia (cervical intraepithelial neoplasia, CIN) [168]. CIN-1 (low-grade) is mild l dysplasia that can regress; it is confined to the lower basal third of the epithelium. CIN-2 (moderate dysplasia affecting the basal two-thirds) and CIN-3 (high-grade or carcinoma-in situ where the entire thickness of the epithelium is dysplastic) can both progress to invasive carcinomas when the basement membrane is breached. The molecular mechanisms HPV uses to transform cervical epithelial cells are known [169]. HPV early genes, E6 and E7, integrate into the host genome leading to a persistent infection that disrupts signalling cascades within the host cell. Hyperproliferation and genomic instability are promoted by E6-dependent degradation of TP53 and the release of the transcription factor, E2F from retinoblastoma protein (RB1) by E7 [170, 171]. Extensive characterisation of genomic alterations in large cohorts has been undertaken. In 228 primary cervical carcinomas, *APOBEC* mutational signatures and novel mutations in *SHKBP1*, *ERBB3*, *CASP8*, *HLA-A* and *TGFBR2* were revealed [172]. Amplifications in immune targets *CD274* (*PD-L1*) and *PDCD1LG2* (*PD-L2*) and the *BCAR4* long non-coding RNA were also observed. Not all cervical tumours are associated with HPV infections and therefore will not be prevented by vaccination. A unique set of these rare HPV-negative cervical carcinomas that resemble endometrial tumours have higher frequencies of *KRAS*, *ARID1A* and *PTEN* mutations.

The nature of the progenitor cells giving rise to ADC and SCC is controversial but adult stem cells can be a source of tumours [173]. They may arise from undifferentiated fetal reserve cells, transdifferentiation of cells of the TZ or even stromal cells [174, 175]. A transcriptomic comparison of cervical organoids with samples of cervical carcinomas revealed shared expression profiles between endocervical organoids with ADC and ectocervical organoids with SCC, suggesting that they may originate from two distinct lineages [78]. Thus, these organoids provide a model system to study carcinogenesis and to identify specific therapeutic targets for each lineage. Patient-derived cervical organoids that represent heterogeneity of the tumour cells are needed to test potential therapeutic targets for cervical carcinomas. There are efforts to establish growth conditions for in vitro culturing of organoids of cervical carcinomas. Indeed, organoids from a rare subtype of cervical cancer, clear cell carcinoma, have been established and can be propagated for 6 months in a medium containing EGF, Rspondin-1, Noggin, ROCK inhibitor and Jagged-1 [176]. Progress in the development of these in vitro tools from all the subtypes of the cervical cancers would be invaluable for drug and immunotherapy testing, as well as for personalised medicine.

### Infection of the FRT

The vagina and cervix are colonised by diverse commensal microorganisms, microbiota, that maintain a symbiotic relationship with the host. These can be outnumbered by pathogens resulting in infection that can ascend to the upper FRT. The most prevalent pathogens include *Treponema pallidum* (syphilis), *Neisseria gonorrhoeae*, *Chlamydia trachomatis*, *Trichomonas vaginalis*, HPV and herpes simplex virus (HSV) [177]. There has been progress in understanding the interactions between the host, the native microbiota and the invading pathogens, and how these affect outcome. Most experimental models to study host-pathogen interactions have used genetically unstable 2D cancer cell lines or immortalised cell lines adapted to in vitro cell culture. Although murine models have also contributed to understanding the human situation, mice have different microbiota and have coevolved with different sets of pathogens and the patterns of the disease vary between the two species [178].

**Fig. 5:**
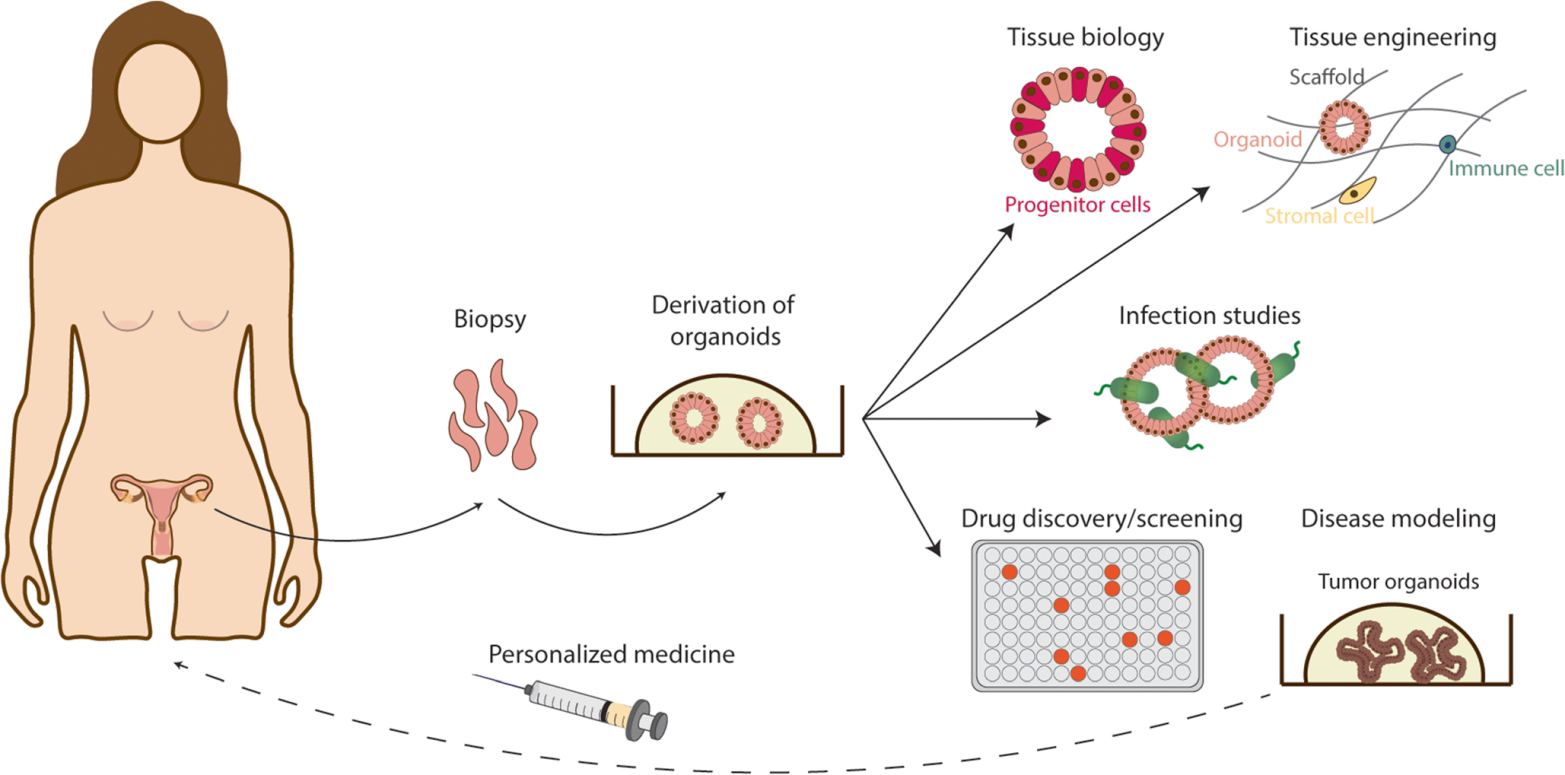
Future applications of organoids of the FRT. Organoids of the FRT are derived from biopsies and can be used to study tissue biology, cell-cell interactions, host-pathogen interactions and disease. Tissue engineering methods combine organoids with other cell types to develop more tissue-like models that include non-epithelial cells. Organoids derived from pathological tissues are useful for drug screening for personalised medicine

Organoids are an excellent alternative to study host-pathogen interactions because the molecular mechanisms mimic normal human physiology. Infection of 3D transwell-based air liquid interface cultures (organotypic cultures) of human squamous ectocervical epithelium demonstrated the ability of Chlamydia to infect differentiated luminal cells with progression towards the basal stem cell compartment caused by disruption of epithelial integrity and induction of epithelial to mesenchymal transition [123]. Chlamydia increases hypermethylation of DNA, which is an indicator of accelerated molecular ageing and might be a contributing factor in the development of tubal pathologies, including the initiation of HGSC. A recent study modelled chronic chlamydia infections using FT organoids and the resulting impact on host DNA methylation [179]. The infected organoids showed higher stemness potential with reduced differentiation mediated by activation leukaemia inhibitory factor (LIF) signalling. HPV fail to replicate in transformed 2D cell lines as they require all stages of epithelial differentiation to replicate and complete their life cycle. Squamous stratified ectocervical organoids can facilitate HPV replication as well as modelling the native viral infection and the long-term impact on the tissue. Although co-infection of FRT is often associated with disease severity for example, HPV and Chlamydia co-infections are linked to increased progression of cervical carcinoma, molecular mechanisms of such co-infections are rarely investigated [87, 88]. Additionally, organoids would allow greater flexibility for manipulation and experimental design to unravel the molecular changes induced by infection. Thus, there is great potential in using the FRT organoids for research into infections of the FRT to model dynamics of host-microbiome interactions, alterations in epithelial homeostasis and repair and progression of pathological changes (Fig. 5).

## Future development and applications of FRT organoids

### Genome editing of FRT organoids using CRISPR-Cas9

Organoid technology is a powerful tool to study tissues in vitro due to its amenability to a range of downstream applications including genome editing. Genetic manipulation of cell lines has been an essential tool to investigate gene function, to immortalise primary cells and to fluorescently label proteins. The discovery and application of the CRISPR (clustered regularly interspaced short palindromic repeats)/Cas9 system have revolutionised the way genome editing can be achieved due to its high specificity, efficiency in the targeting mechanism and simplicity of use. CRISPR/Cas9 is a component of the bacterial adaptive immune system against bacteriophages [180–183]. In genome engineering applications in mammalian cells, the CRISPR/Cas9 system is exploited to generate double-strand breaks in a specific region of interest [184, 185]. This is achieved by a small guide RNA (gRNA), homologous to the sequence of interest, which directs the Cas9 endonuclease to the target site where it cleaves the DNA. The cell repairs this double-strand break either by the error-prone non-homologous end joining (NHEJ) pathway that often results in mutations or by homology-directed repair (HDR) in the presence of a DNA repair template. There are now many variations of this technique that allow the selective activation or repression of gene expression, site-specific epigenetic regulation and genome-wide screens (reviewed in [186, 187]).

Genome editing has been achieved in many different organoid models and is being used to gain insight into tissue biology and disease from investigating the correction of mutations to the role of driver mutations in cancer progression [188–190]. For organoids of the FRT, CRISPR/Cas9 has been applied to FT organoids to study the effects of *TP53* and *RB1* knockout on the development of ovarian carcinoma [107]. Using a similar forward genetics approach, the progression of other FRT cancers can be studied by introducing driver mutations. For example, progression of EC can be modelled by introducing sequential mutations in *PTEN*, *KRAS* and *FGF2* or *ERBB2* in EO and investigating the pro-carcinogenic changes through gene expression profiling and genome sequencing. Emerging studies have shown how FRT organoids can support infections as well as recapitulating chronic infections. For instance, chronic infection of FT organoids with *Chlamydia trachomatis* revealed altered tissue homeostasis and epigenetics [179]. Furthermore, investigation of host-pathogen interactions in combination with CRISPR/Cas9 will allow the identification of host factors involved in the response to infection and genes that may be involved in the malignant transformation of cells. The application of CRISPR/Cas9 technology on FRT organoids is clearly a powerful approach to address many questions in the field of reproduction (Fig. 5). However, as for all targeting approaches, careful validation of the genome-edited organoids must be performed. Furthermore, the efficiency of targeting can be an issue for certain organoid types but new tools are becoming available to overcome these limitations (reviewed in [191]).

### Personalised medicine

Personalised medicine is a therapeutic strategy specifically directed to individual patients and the unique genomic and pathological features of the disease [192]. Many disorders of the FRT are heterogeneous and the ‘one-size-fits-all’ approach is not always effective. However, personalised approaches have been difficult to implement because of the lack of techniques to culture and propagate diseased cells that maintain their cellular composition and behaviour in vitro. The organoid technique can overcome these limitations and recent advancements have shown great promise for personalised medicine approaches to be adopted in the clinic. As discussed earlier, organoids derived from EC and OC are already paving the way for personalised medicine in this field by allowing the propagation of the tumour whilst retaining its cellular heterogeneity on which to perform drug screening to identify effective combination of treatments (Fig. 5) [107, 113]. The ability to cryopreserve FRT organoids provides the added benefit that they can be bio-banked and used for pre-clinical drug screening.

Another area where personalised medicine can make an impact is in assisted reproduction techniques (ART). Although great advancements have been made since the birth of the first in vitro fertilisation (IVF) baby in 1988, currently 60–90% of IVF attempts with a transfer of high quality embryos are unsuccessful resulting in either failure of implantation or miscarriage (loss of pregnancy within 20-week gestation) [193, 194]. These conditions are distressing for the couples and are also a financial burden.

An important underlying issue contributing to IVF failure is the need to synchronise embryonic development with endometrial receptivity [195, 196]. The ‘window of implantation’ (WOI) is at the mid secretory phase when decidualisation commences and there is maximal receptivity. Based on histological studies, this is between (LH, day of LH peak/ovulation) + 6 and LH + 10 in a natural cycle [197]. Compared with normal endometrium at the time of implantation, some IVF patients show signs of premature decidualisation and dyssynchronous differentiation of the glands and stroma [198–200]. Although the changes throughout the cycle in expression of integrins, secretory products and signalling molecules like LIF have been extensively described, the mechanisms regulating receptivity are still unknown [197, 201]. There are still no robust markers to predict the WOI to optimise the timing of embryo transfer. Using EO, the hormonal regimes can be tested to identify optimal protocols for endometrial decidualisation. Furthermore, there is great variation of the menstrual cycle timing between women, so the development of personalised protocols will be of great benefit [202]. However, in order for patient-specific EO to be routinely used as a personalised system in the clinic, development of non-invasive methods to obtain tissue is needed. Currently, biopsies are taken, not ideal for repeated sample recovery from the same patient. Infertility has diverse causes (dysfunction of the ovaries and FT, lesions in the uterus like fibroids or bicornuate uterus, cervical stenosis or poor cervical mucus, squamification of endocervical canal) but is often ‘unexplained’ [203]. These patients may also benefit by deriving organoids for a personalised approach to identify the underlying problems.

### Bioengineering and FRT organoids

Like all in vitro models, organoid systems have limitations and there are still numerous challenges to overcome. An obvious example is the ECM used, generally the commercially available Matrigel, Cultrex, Geltrex or BME, produced by extracting the gelatinous protein mixture from murine Engelbreth-Holm-Swarm sarcomas [204]. Because they are extracted from tumours grown in vivo, there is considerable batch-to-batch variation in growth factors present, composition of ECM and stiffness of the gel that have effects on cell behaviour [205, 206]. Generation of chemically defined and reproducible hydrogels are clearly needed and these will also reduce costs and use of animals. Furthermore, to use organoids for transplantation, avoidance of ECM derived from animals that might be immunogenic and carry pathogens is essential. Intestinal organoids can now be grown in synthetic hydrogels with known concentrations of ECM components, collagen and laminin, or the use of short RGD peptides, the adhesion motif for integrins [207–209]. Such studies are beginning to be established also for FRT organoids [210]. It is clear that the ECM composition and stiffness differ throughout the FRT and this must also be taken into account. Indeed, endometrial stiffness varies across the cycle and in pregnancy [211]. More information about the normal composition and stiffness of the ECM in the whole FRT will allow introduction of tailored synthetic matrices for the different organoid types.

Tissue-derived organoids are composed of epithelial populations and lack stromal, immune and nerve cells as well as blood vessels. Although this reductionist approach is an advantage for the study of epithelial behaviour, it is a limitation when investigating mucosal function in vivo that may be controlled through cell-cell interactions. For example, the transition of the squamo-columnar epithelium of cervix is controlled by opposing Wnt signals provided by the underlying stromal tissue and alteration to these signals reshape the epithelial homeostasis including metaplastic adaptations [78]. Increased Wnt antagonists in the endocervix lined by columnar epithelium and uterus promote the development of preneoplastic squamous metaplasia [78]. Endometrial stromal-epithelial interactions are particularly important in the response to ovarian hormones when stromal cells respond to P4 by secreting growth factors and cytokines (e.g. prolactin, IGFBP-1, IL-11) that stimulate the differentiation of epithelial cells [65, 66]. Studying these interactions in co-culture models of endometriosis where the stromal component probably plays a key role in driving pathogenesis will be important [103, 212–214]. Introduction of immune and stromal cells to organoid models will allow investigation of their roles in tissue homeostasis and function as well as infections with chlamydia, gonorrhoea, tuberculosis or HPV. In addition, to model the complex microenvironment of tumours of the FRT, it will be necessary to introduce non-malignant cells.

Different co-culture methods to study cell-cell interactions have been adopted by introducing stromal or immune cells directly in the ECM droplet together with the organoids (reviewed in [215]). Other methods include organotypic cultures in which stromal cells (primary or cell lines) are grown as a monolayer, either on an ECM-coated surface or in a cell culture insert, and then seeded with epithelial cells. This does recapitulate essential features of tissues and has been widely used for the study of the cervix and other stratified tissues [216, 217]. Bioengineered 3D scaffolds, usually made with collagen, decellularised tissue or other biomaterials, can also be used to achieve a more organised tissue (reviewed in [218]). Co-culture of EO with primary endometrial stromal cells in a collagen I scaffold has been reported [219]. The epithelial cells differentiate into ciliated and secretory cells and both epithelial and stromal cells respond to E2 and P4 by secreting typical proteins associated with decidualisation. The advantage of this system is that the organoid secretions, which are normally concentrated within the lumen, are more accessible. Glandular structures are still absent in this model; however, it only consists of a single epithelial layer with underlying stroma. A similar approach to how intestinal crypts have been modelled by using a microengineered scaffold that guided the growth of primary intestinal cells to recapitulate crypt-villus organisation may be helpful [220]. Emerging genomic technologies such as single-cell RNA sequencing and spatial transcriptomics will provide an important framework to guide the generation of tissue-like models [120].

Microfluidic systems have also been used to model tissues in an ‘organ-on-a-chip’ approach. A microfluidics device is a microchip, with channels moulded into a material to confine fluid movement. The material that is commonly used for culturing cells is poly-di-methyl siloxane which can be moulded and is biocompatible and permeable to gas. The main advantage of microfluidic systems is the ability to accurately control the experimental environment allowing for continuous flow of media and generation of chemical gradients at a small scale. Furthermore, sensors can be inserted within the system so specific measurements can be monitored in real-time. A microfluidic platform that models the entire FRT is described that uses primary cells to study the endocrine loops between the various tissues [221]. In order to test the functionality of this system, maturation and responses of murine follicles were shown. Similarly, lung and kidney organoids in such a system allows the study of interactions between multiple cell types [222, 223]. Challenges remain, however, including the optimisation of media for different cells, long-term culture and reproducibility. The application of these models to the FRT will be essential to address many outstanding questions (Fig. 5).

### Organoids to study early pregnancy

The development of FRT organoids together with the recent advancements in human embryo culture and stem cell-derived synthetic embryo models provide a unique opportunity to study the black box of early human development [224–226]. The outer trophectoderm layer of the blastocyst rapidly establishes contact with the endometrium, transformed into decidua during pregnancy. Besides providing the site of implantation, secretions from the luminal epithelium are essential for implantation by providing growth factors and exosomes [227, 228]. These maternal-fetal interactions in humans have not been possible to study ex vivo to date. The derivation of FT and EO now presents an opportunity to dissect these signals and to study implantation ‘in a dish’ by co-culture with natural or synthetic embryos. As technologies develop and push the boundaries of what is possible to achieve in the laboratory, it is of central importance to also consider the ethical issues [229, 230].

Upon implantation, the embryo burrows into the decidua and the formation of the placenta begins. The next stages of the development of the placenta are also regulated by maternal signals through decidual glandular secretions [64]. However, the exact nature of these secretions and their effect on the major cell type of the placenta, the trophoblast, are unknown. These maternal-fetal interactions are bidirectional, as signals from the developing placenta also stimulate the decidua in a positive feedback loop [47]. This was demonstrated when human EO were stimulated with placental products, human chorionic gonadotropin (HCG) and placental lactogen, they differentiated further resembling ‘pregnant’ decidual glands [50].

The establishment of long-term, genetically stable organoids of the early human placenta provides an experimental model to study these early events in vitro [231, 232]. Human placentation is particularly invasive as the extravillous trophoblast (EVT) of the placenta detach from the villi and migrate through the decidua to the inner third of the myometrium [233]. EVT transform the decidual spiral arteries to allow maximal gaseous/nutrient exchange at the fetal-maternal interface and this is essential for pregnancy as the major disorders (pre-eclampsia, miscarriage and still-birth) show defects in this process [234]. There is little understanding of the molecular and cellular signals between the decidua and placenta that regulate this complex process. Trophoblast organoids can differentiate into the two main trophoblast subtypes: under proliferative conditions, they grow as villous structures with Ki67+, CDH1+, EpCAM+ mononuclear villous trophoblast on the outside and the multinucleated syncytiotrophoblast in the inside. Invasive, human leukocyte antigen-G+ (HLA-G+) EVT appear either when Wnt signals are omitted or by using differentiation medium for 2D human trophoblast stem cells [235]. They also recapitulate key placental functions: the syncytiotrophoblast secretes placental products identified by mass spectrometry, as well as resulting in a positive pregnancy stick. EVT are vigorously invasive, captured by time-lapse microscopy [231]. The effect of the decidual and placental signals could be investigated systematically using endometrial and trophoblast organoids and stimulating them with recombinant proteins of factors identified from their secretome. The ultimate goal is to co-culture the two organoid types together to establish a model of the maternal-fetal interface.

## Conclusions

The FRT undergoes dramatic changes throughout a woman’s life and its proper function is essential for reproductive health and wellbeing. Research in this field has been hampered by the lack of physiologically relevant models together with justifiable ethical concerns. The generation of organoids of the FRT that can model both healthy and diseased tissues will be transformative. The ability of organoids to recapitulate key anatomical, molecular and functional features of tissues as well as being amenable to many downstream applications makes them a powerful model system. Organoids are more expensive and labour intensive than 2D cell lines but the technique is relatively simple and can be easily adopted by other labs. As we move forward with these tools, it is imperative that we also continue to address issues including reproducibility and thorough comparisons with tissues to benchmark and improve the organoid models [120, 236]. Furthermore, interdisciplinary approaches such as bioengineering to incorporate multiple cell types will help further develop these models. FRT organoids will allow us to investigate the fundamental biology of how these tissues function, how the different cell types interact, as well as gaining insight into the pathogenesis of cancer and infectious diseases. They also provide translational opportunities in drug discovery and personalised medicine. These emerging tools open up new possibilities to address outstanding questions in the research field of the FRT, thereby providing great promise for improving the health and wellbeing of women.

## Abbreviations

3D: three-dimensional
ADC: adenocarcinoma of the cervix
BME: basement membrane extract
BMP: bone morphogenetic protein
cAMP: cyclic adenosine 3′,5′-cyclic monophosphate
CDH1: Cadherin 1
CIN: cervical intraepithelial neoplasia
CL: corpus luteum
CRISPR: clustered regularly interspaced short palindromic repeats
E2: oestrogen
EC: endometrial carcinoma
ECM: extracellular matrix
ECT: ectopic endometrium
EGF: epidermal growth factor
EMT: epithelial-to-mesenchymal transition
EpCAM: epithelial cell adhesion molecule
EO: endometrial organoids
ESR1: oestrogen receptor α
EUT: eutopic endometrium
EVT: extravillous trophoblast
FGF: fibroblast growth factor
FRT: female reproductive tract
FSH: follicle stimulating hormone
FT: fallopian tube
HGSC: high-grade serous carcinoma
HPO: hypothalamus-pituitary-ovarian (axis)
HPV: human papillomavirus
HSV: herpes simplex virus
IGFBP: insulin-like growth factor binding protein
IVF: in vitro fertilisation
LGR5: leucine-rich repeat-containing G-protein coupled receptor 5
LH: luteinizing hormone
MAPK: mitogen-activated protein kinase
MMP: matrix metallopeptidases
OC: ovarian cancer
OSE: ovarian surface epithelium
P4: progesterone
PGE2: prostaglandin E2
PIK3CA: phosphatidylinositol-4,5-Bisphosphate 3-Kinase
PGR: progesterone receptor
PSC: pluripotent stem cells
PTEN: Phosphatase and tensin homologue
ROCK: Rho-associated protein kinase
SCC: squamous cell carcinoma of the cervix
SCJ: squamocolumnar junction
SSEA1: stage-specific embryonic antigen-1
TBF-β: transforming growth factor beta
TZ: transition zone
Wnt: wingless-related integration site
